# The Functional State of Hormone-Sensitive Adenylyl Cyclase Signaling System in Diabetes Mellitus

**DOI:** 10.1155/2013/594213

**Published:** 2013-09-28

**Authors:** Alexander O. Shpakov, Kira V. Derkach

**Affiliations:** Sechenov Institute of Evolutionary Physiology and Biochemistry, Russian Academy of Sciences, Thorez Avenue 44, Saint Petersburg 194223, Russia

## Abstract

Diabetes mellitus (DM) induces a large number of diseases of the nervous, cardiovascular, and some other systems of the organism. One of the main causes of the diseases is the changes in the functional activity of hormonal signaling systems which lead to the alterations and abnormalities of the cellular processes and contribute to triggering and developing many DM complications. The key role in the control of physiological and biochemical processes belongs to the adenylyl cyclase (AC) signaling system, sensitive to biogenic amines and polypeptide hormones. The review is devoted to the changes in the GPCR-G protein-AC system in the brain, heart, skeletal muscles, liver, and the adipose tissue in experimental and human DM of the types 1 and 2 and also to the role of the changes in AC signaling in the pathogenesis and etiology of DM and its complications. It is shown that the changes of the functional state of hormone-sensitive AC system are dependent to a large extent on the type and duration of DM and in experimental DM on the model of the disease. The degree of alterations and abnormalities of AC signaling pathways correlates very well with the severity of DM and its complications.

## 1. Introduction 

Diabetes mellitus (DM) is a major global health problem affecting, according to the World Health Organization, more than 346 million people worldwide [1]. It is one of the most severe metabolic disorders in humans characterized by hyperglycemia due to a relative or an absolute deficiency of insulin or the resistance of target tissue to regulatory action of the hormone, or both. Type 1, insulin-dependent and type 2, non-insulin-dependent DM (T1DM1 and T2DM2) both induce a large number of diseases and dysfunctions of the nervous, cardiovascular, excretory, reproductive, and other systems of the organism [2–9]. Severe complications of DM are found in more than a quarter of diabetic patients. A new view of the nature and pathogenesis of DM-induced complications shared by many specialists nowadays has been prompted by the study of functional activity of hormonal signaling systems regulated by insulin, insulin-like growth factor-1 (IGF-1), and leptin, the principal players responsible for the development of DM and its central and peripheral complications, and by a wide spectrum of other hormones and neurotransmitters, including biogenic amines, purines, glutamate, peptide, and glycoprotein hormones controlling the fundamental cellular processes. The data were obtained showing that the alterations and abnormalities of functional activity of these systems and the changes of expression of hormones and signal proteins in DM led to the disturbances of growth, differentiation, metabolism, and apoptosis in different types of cells and contributed to triggering and developing the pathological processes in the organs and tissues of diabetic individuals [10–15]. 

It is a common knowledge that in the regulation of physiological and biochemical processes the most important role belongs to a hormone-sensitive adenylyl cyclase (AC) signaling system. Activating or, alternatively, inhibiting this system, many hormones, growth factors, and hormone-like substances control the activity of cAMP-dependent signaling cascades and transcription factors and thus regulate gene expression and the other cell processes. The AC system has three main components: (i) G protein-coupled receptor (GPCR) of the serpentine type that recognizes and specifically interacts with hormone, (ii) the *αβγ*-heterotrimeric G protein of the stimulatory (G_s_) and inhibitory (G_i_) types, and (iii) the enzyme adenylyl cyclase (AC) catalyzing the formation of cAMP, a second messenger. The system specifically recognizes a hormonal signal, amplifies it, and transmits to the intracellular cAMP-dependent effector proteins. The GPCR acts as a molecular switch that provides the transduction of hormonal signals from extracellular ligand-binding site to the G proteins located at the cytoplasmic side of the plasma membrane. Ligand-activated GPCR induces the conformational changes in the GDP-bound G*α* subunit, leading to GTP exchange for GDP. G*α*-GTP then dissociates from G*βγ*-dimer complex, and G*α*-GTP and G*βγ* both interact with the enzyme AC, stimulating or inhibiting its activity. 

The present review is devoted to the alterations and abnormalities in the GPCR-G protein-AC signaling system regulated by biogenic amines and polypeptide hormones in the brain, heart, skeletal muscles, liver, and the adipose tissue in experimental and human DM and to the role of the changes in AC signaling cascades in the pathogenesis and etiology of DM and its complications. 

## 2. cAMP Signaling in the Brain

Many neurodegenerative disorders, diabetic encephalopathy and Alzheimer's disease in particular, are associated with DM. In diabetic patients, they are manifested as alterations in neurotransmission, electrophysiological abnormalities, structural changes, and cognitive deficit [16]. Since the brain is mainly a glucose-dependent organ, a severe hyperglycemia in T1DM, mild hyperglycemia typical of T2DM, and recurrent hypoglycemia induced by inappropriate insulin therapy are the major factors responsible for the development of DM-induced cerebral disorders [17]. In the diabetic brain, a large number of AC signaling cascades regulated by different hormones and neurotransmitters and forming a well-coordinated signaling network undergo significant changes due to disturbances in these cascades and abnormalities in overall neuronal signaling, including both cAMP-dependent and -independent pathways [12–14, 18–26]. Currently available data on the hormonal signaling in the diabetic brain indicate that the most essential changes occur in AC signaling cascades regulated by biogenic amines, such as dopamine (DA), norepinephrine (NE), and serotonin (5-hydroxytryptamine, 5-HT) and by peptide hormones and adenosine. 

DA is the predominant catecholamine neurotransmitter in the brain, which controls a variety of CNS functions (locomotor activity, cognition, emotion, food intake, and neuroendocrine regulation) and plays multiple roles in the periphery as a modulator of cardiovascular function, hormone secretion, and gastrointestinal motility. DA activates AC via G_s_-coupled DA receptors of types 1 and 5 (DA_1_R and DA_5_R) and inhibits the enzyme via G_i_-coupled DA_2_R, DA_3_R, and DA_4_R. It was found experimentally that in the brain of diabetic animals the dopaminergic signaling was altered, mainly due to the changes at the initial stages of DA-induced signal transduction which involves DARs, G_i_, or G_s_ proteins and their effectors, AC in particular, and these alterations are brain region specific [27]. In the cerebral cortex of rats with T1DM induced by streptozotocin (STZ) treatment, the expression of DA_1_R and DA_2_R and the total DAR binding were increased; in the cerebellum, DA_1_R was downregulated and DA_2_R upregulated, a total number of DARs being however decreased, and in the hypothalamus and brainstem a significant decrease in the DA content and the number of DA_2_Rs and an increase in DA_2_R affinity were shown [22, 28]. The treatment with insulin partially restored DM-induced changes of DA_1_R and DA_2_R expression and DA_2_R receptor binding parameters in the diabetic brain to near control, thereby improving the cognitive and emotional functions impaired in T1DM [28, 29]. The alteration of DA-induced signaling in the diabetic brain is associated with their downstream components, such as cAMP response element binding protein CREB, the important transcription factor, playing a pivotal role in DAR-mediated nuclear signaling and neuroplasticity [30]. It was found that STZ-induced DM produces a significant attenuation of functional activity of CREB in the cerebral cortex and cerebellum of diabetic rats and these alterations are eliminated by the insulin treatment [22]. 

We showed that, in the brain of rats with one-month T1DM, induced by STZ at a dose 65 mg/kg of body weight, and with prolonged, 7-month, T1DM, induced by three consecutive injections of STZ on the first, 10th, and 80th days of experiments at dropped doses 40, 35, and 30 mg/kg, the AC stimulating effect of DA realized via G_s_-coupled DA_1/5_Rs did not change significantly [31, 32], and, in the brain of rats with the neonatal model of T2DM, duration 3 to 6 months, this effect even increased a little [33]. The AC inhibitory effect of DA_2_R agonist bromocriptine and its stimulating effect on 5′ guanylylimidodiphosphate (GppNHp) binding of G_i_ proteins in the synaptosomal membranes isolated from the brain of diabetic rats were decreased, predominantly in T1DM [32, 34, 35]. As the binding characteristics of DARs and the catalytic activity of AC did not change essentially, a suggestion was made that the impairment of bromocriptine-induced AC inhibition in the diabetic brain was due to the reduced function of G_i_ proteins [36]. A similar assumption was made by the other authors. In their view, the attenuation of DA_2_R agonist-induced suppression of appetite in STZ rats might be the result of reduction of G_i_ protein activity in the diabetic brain [37]. 

The brain serotonergic signaling is involved in the regulation of feeding, locomotion, reproduction, sleep, pain, aggression, and stress responses, as well as thermogenesis, cardiovascular control, circadian rhythm, and pancreatic function. The abnormalities of 5-HT-regulated signaling cascades in the diabetic brain provoke disturbances in neuronal processing and the alteration of plasticity of neurotransmission and play an important role in DM-induced CNS disorders [38, 39]. This is caused first of all by the alterations of the brain sensitivity to 5-HT, which depends on the functioning of 5-HT-regulated cascades and the decrease of cerebral concentrations of 5-HT and its metabolites as a result of the impairment of biochemical conversion, reuptake and transport of 5-HT. It was shown that 5-HT-regulated AC signaling pathways including G_i_-coupled 5-hydroxytryptamine receptor of the type 1 (5-HT_1_R) were altered significantly. One-week STZ-induced DM markedly reduced the flat body posture induced by 5-HT_1A_R agonist 8-hydroxy-2-(dipropylamino) tetralin hydrobromide (8-OH-DPAT), which indicated that STZ-induced T1DM profoundly affected the sensitivity to drugs acting via 5-HT_1A_R [40]. 

In our experiments, no alterations of the sensitivity of AC system in the brain of STZ rats to selective agonists of G_s_-coupled 5-HT_6_R were detected, while AC sensitivity to agonists of G_i_-coupled 5-HT_1A_R and 5-HT_1B_R was decreased significantly [32, 33, 35, 41]. In the brain of rats with prolonged T1DM and neonatal T2DM, the AC inhibitory effects of 5-HT_1B_R-agonist 5-nonyloxytryptamine were reduced by 79 and 70%, respectively. The increase of GppNHp binding of G proteins induced by 5-nonyloxytryptamine and nonselective 5-HT_1_R agonist 5-methoxy-*N*,*N*-dimethyltryptamine in the case of the neonatal model of T2DM was reduced by 57 and 51%, respectively [35, 41]. In our view, the weakening of 5-HT_1_R-mediated signaling may be associated with a decrease of expression and activity of G_i_ proteins, because in the diabetic brain the impairment of other G_i_-coupled cascades was also detected. 

Due to their ability to regulate the synaptic mechanisms, NE and other ligands of adrenergic receptors (ARs) are essential modulators of memory. The activation of *β*-ARs by NE leads to facilitation of the synaptic transmission through the mechanism involving the increase of the level of intracellular cAMP and protein synthesis *de novo*, thus contributing to the memory acquisition and maintenance [42]. It was found that the population of G_s_-coupled *β*-ARs in the regional brain of *db/db* mice was depressed, which demonstrates a considerable modification of adrenergic signaling [43]. The level of NE and the number of G_i/o_-coupled *α*_2_-ARs in the frontal cortex, septal area, amygdala, hypothalamus, and medulla of 4–16-week-old *db/db* mice with T2DM-like state were, on the contrary, elevated compared to those of control animals and this increase correlated with the changes in blood glucose level and body weight [43]. The alteration of adrenergic signaling in the brain of *db/db* mice, the genetic model of T2DM, may be causally associated with the consequences of the changes in structural integrity and neuronal competence of the medial basal hypothalamus. The alterations in the brain adrenergic signaling were also shown in STZ-induced T1DM. In the brainstem of STZ-treated rats, the content of epinephrine and the NE/epinephrine turnover were increased, and the affinity of *α*_2_-ARs to selective agonists was significantly reduced [44]. Insulin treatment induced the decrease of the NE content and the restoration of the binding parameters of *α*_2_-ARs to normal values. In the hypothalamus, thalamus, and amygdala of STZ rats, the density of *β*_1_-, but not of *β*_2_-, ARs was increased, and the rate of metabolism of NE was decreased [45]. 

The neurodegenerative processes in the diabetic brain are largely associated with the damage in the cerebral microvessels whose functions are controlled via different signaling systems, the adrenergic in particular. The number of *β*-ARs in the cerebral microvessels of Zucker diabetic fatty (*fa/fa*) rats and STZ rats was significantly decreased despite the receptor affinity did not change [46]. The isoproterenol-stimulated AC activity in the cerebral microvessels of diabetic rats was significantly lower compared with control, giving evidence for the weakening of *β*-AR-mediated regulation of cAMP-dependent signaling in T1DM [47]. We showed the decrease of the AC stimulating effect of isoproterenol in the brain of rats with prolonged T1DM induced by three consequent injections of moderate doses of STZ [32]. These results suggest that alterations of central adrenergic regulation in small vessels may be involved in microvasculature disturbances in the diabetic brain. Note that in the cerebral cortex, striatum, and hippocampus of rats with the neonatal model of T2DM the AC stimulating effect of isoproterenol had no changes [35]. It follows that the alterations of adrenergic signaling are brain region specific and depend to a great extent on the DM model, glycemic control, and duration of DM. 

In DM, there are significant changes in the brain AC system regulated by peptide hormones and neurotransmitters, such as melanocortin, glucagon-like peptide-1 (GLP-1), somatostatin, relaxin, and pituitary AC-activating polypeptide (PACAP). In human and experimental T2DM, the gene expression of melanocortin receptor of type 4 (MC_4_R) coupled with AC via G_s_ proteins in the brain was restricted, and MC_4_R-mediated signaling pathways were significantly reduced [48]. It is generally accepted that MC_4_R-agonists *α*-melanocyte-stimulating hormone (*α*-MSH) and melanotan II promote a negative energy balance by decreasing the food intake and increasing the brain activity and energy expenditure, whereas hypothalamic agouti-related peptide (AgRP), MC_4_R antagonist, on the contrary, increases food intake [49]. Along with this, MC_4_R pathways regulate glucose metabolism and insulin sensitivity [50–52]. Central injection of MC_4_R-agonists reduces insulin secretion, while administration of MC_4_R-antagonists increases plasma insulin level. Indeed, elevated insulin level and impaired insulin sensitivity were detected in the young lean MC_4_R knockout mice, even before the development of pronounced hyperphagia and obesity [50, 53]. The mice with functionally inactive MC_4_R had obesity strikingly reminiscent of the agouti syndrome, which indicates that the disturbances in MC_4_R signaling pathways were the primary cause of the agouti obesity. The hypothalamic melanocortin system controlling adiposity levels rapidly and more efficiently than the other central signaling systems is regulated by leptin [52]. The decrease of leptin level due to fasting or genetic leptin deficiency led to the reduced expression of proopiomelanocortin gene and to the increased expression of AgRP gene [54], and leptin infusion, on the contrary, increased the hypothalamic content of proopiomelanocortin and MC_4_R and inhibited the production of AgRP [55]. Since MC_4_Rs are involved in neuroprotective action, regeneration and promotion of adaptive plasticity of neuronal and glial cells [56, 57], the alterations and abnormalities of hypothalamic melanocortin-sensitive AC system in the hypothalamus are the prime causes of neurodegenerative processes in the diabetic brain. The positive regulatory effects of *α*-MSH and selective MC_4_R-agonists on neuronal plasticity and survival could be mediated by their influence on neuronal signaling regulated by other neurotransmitters, GABA in particular [58]. 

The contribution of the central melanocortin system in etiology and pathogenesis of T2DM and obesity is fully confirmed by the data on the immunization of rats with peptides corresponding to the N-terminal extracellular domain MC_4_R and to the first and third extracellular loops of MC_3_R [59, 60]. In rats injected with peptide corresponding to the N-terminal domain of MC_4_R, like in the case of the blockade of hypothalamic MCRs by antagonists, the food intake, body weight, plasma insulin, and triglycerides levels increased significantly [59]. The antibodies against the N-terminal domain of MC_4_R acted as partial MC_4_R agonists and decreased cAMP level in cell cultures. Antibodies against peptide derived from the first loop of MC_3_R amplified the AC stimulating effect of *α*-MSH, while antibodies against peptides, the derivatives of the third loop of the same receptor, reduced the effect of hormone, acting as a noncompetitive MC_3_R antagonist. In rats injected with peptide from the third intracellular loop of MC_3_R, the body weight and blood pressure were increased, and the motor activity was decreased. The plasma levels of triglycerides, insulin, and leptin were much higher compared with control, the same as in T2DM. But the rats injected with peptide from the first loop had no changes of physiological and biochemical parameters [60]. This is the evidence that peptides derived from the MCRs and the antibodies against these peptides directly influence melanocortin signaling pathways and cause changes in the brain signaling network. Their action being receptor and site specific, they can induce either inhibition or enhancement of signal transduction via the cognate receptor. This is in good agreement with the results obtained with synthetic peptides derived from the extracellular and intracellular regions of different GPCRs [61, 62]. Thus, peptides corresponding to the extracellular loops of MCRs are a promising tool in the study of etiology of DM and its cerebral complications and give a perspective approach to develop new models of DM and obesity based on antibody-induced deregulation of the brain AC signaling. 

In the brain, GLP-1 via specific receptors activates AC and intracellular cAMP-dependent pathways, modulates Ca^2+^ channels and other effector systems, and functions as a neurotransmitter. GLP-1 has the growth factor-like and neuroprotective properties and controls learning, memory, and synaptic plasticity [63–65]. In the periphery, GLP-1 is responsible for modulating blood glucose concentrations by stimulating glucose-dependent insulin secretion and by activating *β*-cell proliferation. GLP-1 is effective in restoring first-phase insulin response and lowering hyperglycemia in T2DM [66]. GLP-1 receptor agonists, exendin-4, and liraglutide, like the inhibitors of GLP-1 degradation (dipeptidyl peptidase IV inhibitors), have been approved for the treatment of T2DM [67, 68]. Liraglutide, analog of human GLP-1 with prolonged half-life having a fatty acid palmitoyl group conjugated to the side-chain of Lys^26^ and an Arg^34^Ser substitution, is now widely used in T2DM therapy [67]. Exendin-4 and liraglutide injected subcutaneously for 4, 6, or 10 weeks once daily in *ob/ob*, *db/db*, and high-fat-diet-fed mice enhanced proliferation rate of progenitor cells by 100–150% and stimulated differentiation into neurons in the dentate gyrus [65]. The GLP-1 receptor antagonist exendin (9–36) significantly reduced progenitor cell proliferation in these mice. Exendin-4, liraglutide, and GLP-1 analog with Ala^8^Val substitution enhanced long-term potentiation in the brain and reduced the number of amyloid dense-core plaques in mice with insulin resistance and in patients with T2DM-associated obesity and Alzheimer's disease [69]. These results give grounds to say that the GLP-1 analogs show promise in the treatment of T2DM-induced neurodegenerative diseases, because they cross the blood-brain barrier and increase neurogenesis. GLP-1 analogs elicit the insulinotropic activity and improve the central and peripheral symptoms of T2DM [70]. 

The PACAP is a bioactive peptide with diverse activities in the nervous system; it functions as a neuromodulator and neurotrophic factor [71, 72]. PACAP has neuroprotective effect *in vitro* and *in vivo* in ischemia, neurodegenerative diseases and retinal degeneration [73, 74]. PACAP protects neuronal cells from diabetic lipotoxicity, as is shown in the lipotoxicity model induced by the treatment of adult neuronal stem cells isolated from the adult mouse brain subventricular zone with palmitate promoting lipoapoptosis and in the subventricular zone/striatum isolated from obese *ob/ob* mice [75]. Palmitate treatment led to an increase of expression of PACAP receptors, such as PAC-1 and VPAC-2, in the neuronal cells. In the subventricular zone of *ob/ob* mice, the level of expression of PAC-1, VPAC-1, and VPAC-2 receptors was also significantly increased. These data speak in favor of the fact that PACAP counteracted the lipotoxicity, acting via PAC-1 and other types of PACAP receptors, and protected neuronal cells from apoptosis in the diabetic brain, which suggests a potential therapeutic role of PACAP receptor agonists in the treatment of neurological complications induced by T2DM and obesity [75]. 

As was found in our experiments, in the brain tissue of rats with prolonged STZ-induced T1DM, the stimulating effects of PACAP-38, the predominant form of PACAP in mammals, on AC activity and GppNHp binding were significantly decreased and could be largely restored by the long-term treatment with intranasal insulin [32]. In prolonged neonatal T2DM, the AC stimulating effect of PACAP-38 in the synaptosomal membranes was changed a little in the course of three and six months of the disease but decreased significantly in the case of 18-month T2DM [41]. In addition to its important role in the protection of neuronal cells from damage and neurodegenerative changes, the decrease of functional activity of PACAP-38-regulated AC system in the brain in T1DM and T2DM points to abnormalities in PACAP-mediated neuroprotection in DM. 

In the brain, neuropeptide somatostatin specifically binds to five types of G_i_-coupled somatostatin receptors (SSTRs) that are widely expressed in the brain with a distinct distribution pattern [76]. The central signaling pathways of somatostatin and its synthetic analogs are involved in the regulation of secretion of many pituitary hormones, for example, growth hormone, prolactin, and thyroid stimulating and adrenocorticotropic hormones. Somatostatin controls endocrine, sympathetic, behavioral, and visceral responses to stress, cognition, thermogenesis, food behavior, and tumourgenesis of nervous and peripheral tissues [77–80]. We showed a significant decrease in the functioning of somatostatin-regulated AC system in the brain of rats with STZ T1DM and the neonatal model of T2DM [31, 32, 35, 41]. The inhibitory effect of somatostatin on forskolin-stimulated AC activity in the synaptosomal membranes and its stimulatory effect on GppNHp binding capacity of G_i_ proteins in the diabetic brain were markedly reduced, as compared to control, but the therapy with intranasal insulin partially restored both effects. In prolonged, 18-month neonatal T2DM, the AC inhibitory effect of somatostatin was decreased by 33% compared with the nondiabetic rats of the same age, while, in the case of three- and six-month DM, this effect decreased only by 11% and 21% [41]. This gives evidence that with elongation of T2DM the alterations of somatostatin-mediated AC signaling in the diabetic brain are progressively strengthening. 

We regard the decrease of expression and functional activity of G_i_ proteins as one of the main causes responsible for the reduction of AC regulatory effects of somatostatin. At the same time, the reduced number of SSTRs can also contribute to the decrease of somatostatin signaling. The expression of some types of SSTRs was found to be decreased in the hypothalamus and pituitary of rats with T1DM [81]. The mRNA expression of genes encoding SST_1_R, SST_2_R, SST_3_R, and SST_5_R, in the diabetic pituitary, was reduced by 50–80%, and the level of SST_1_R and SST_5_R, restored by insulin treatment. The level of SST_5_R was reduced by 30% in the hypothalamus of STZ rats and completely restored by insulin. Taking into account the importance and complexity of the physiological and biochemical effects of somatostatin and its signaling cascades plus their involvement in the pathogenesis of a variety of neurodegenerative diseases, it would be right to say that the disturbances in somatostatin-sensitive AC system are likely to be responsible for the development of DM-induced CNS complications. 

According to the literature data, there are types of AC whose expression and functional activity in T2DM undergo changes along with the receptor and transducing components of AC system. Using real-time RT-PCR, it was shown that the expression of the type 3 AC (AC3) in the striatum and hypothalamus of Goto-Kakizaki rats, a spontaneous animal model of T2DM, was higher compared with Wistar rats, and the 15-day treatment of Goto-Kakizaki rats with insulin led to a decrease of the enzyme expression [24]. It was shown that dynamics of alterations of AC3 expression in the striatum and hypothalamus and in the pancreatic islets of diabetic rats was similar, which indicates a high probability of the involvement of AC3 in the glucose homeostasis regulation mediated both by CNS mechanisms and insulin secretory activity of pancreatic islets. 

## 3. cAMP Signaling in the Cardiovascular System

DM is well known for its cardiovascular complications including acute myocardial infarction, congestive heart failure, and other manifestations of atherosclerosis and often termed as diabetic cardiomyopathy [10, 82]. Hyperglycemia and insulin deficit in T1DM and hyperinsulinemia and insulin resistance in T2DM induce changes in the contractile function and duration of action potential [83, 84], and these changes are closely linked to the alterations and abnormalities in the adrenergic, purinergic, and other signaling systems and their downstream effectors in the cardiac myocytes [10, 85–87]. In the heart, many effects of the biogenic amines, purines, polypeptide hormones, and other signal molecules are realized via AC signaling system that is implicated in hormonal regulation of arterial tone, reactivity, and cell proliferation. 

The adrenergic signaling, which has a key role in the regulation of the cardiovascular system, changes to the greatest extent in DM. In the cardiac tissue, there are three pharmacologically distinct subtypes of *β*-adrenergic receptors (*β*-ARs). *β*_1_- and *β*_2_-ARs stimulate AC activity via G_s_ proteins, and as a result, the intracellular cAMP level increased, and the activity of protein kinase A (PKA) is enhanced. This leads to phosphorylation of L-type calcium channels, phospholamban, troponin, and ryanodine receptors, all being essential for cardiac function, the control of heart rate, and contractility in particular. *β*_3_-AR is involved in the regulation of cardiac contractility through activation of G_i_ proteins and NO synthase signaling pathway and the stimulation of *β*_3_-AR, unlike that of *β*_1_- and *β*_2_-ARs, which decreases the contractile force in human ventricular muscle [88, 89]. 

In the heart of rats with experimental T1DM, the mRNA and protein levels of *β*-AR subtypes and their functional activity and role in the regulation of heart rate and contractility were changed, and these alterations strongly progressed with increasing duration of DM. In the hearts of rats with STZ-induced T1DM duration of 6 to 14 weeks the expression of *β*_1_-AR and the density of *β*-ARs were decreased significantly compared with control animals [86, 90–93]. At the same time, the mRNA level encoding *β*_2_-AR in the heart of rats with 14-week STZ T1DM was increased, but the density of *β*_2_-AR protein was, on the contrary, decreased which can be attributed to the increased rate of *β*_2_-AR degradation and posttranslational modifications preventing the translocation of this receptor from the endoplasmic reticulum to the plasma membrane [86]. Insulin therapy prevented the reduction of the number of myocardial *β*-ARs and the level of *β*_1_-AR mRNA observed in 6-week STZ T1DM [93]. The increase of the number of myocardial *β*-ARs to near or above control level was detected in the case of treatment of diabetic rats both with insulin and thyroid hormones [94]. 

The mRNA and protein level of cardiac *β*_3_-AR in rats with 14-week STZ DM were twice those in control [86]. The estimated ratio of *β*_1_-, *β*_2_- and *β*_3_-ARs in the heart of control and diabetic rats was 62 : 30 : 8 and 40 : 36 : 23, respectively, and the two-week insulin treatment of diabetic animals increased *β*_1_- and *β*_2_-AR contents, decreased the number of *β*_3_-ARs and, as a result, restored the ratio to 57 : 33 : 10. Note that the left ventricular cardiomyocytes samples from 29 failing human hearts showed a 2- to 3-fold increase of *β*_3_-AR density and a similar increase in the expression of *β*_3_-AR-coupled G*α*_i2_ subunit mediating *β*_3_-AR agonist-induced inhibition of AC activity, as compared to nonfailing human hearts [95]. There are data stating that the molecular variations of *β*_3_-AR lead to obesity, insulin resistance, and T2DM [96]. It was shown also that a mutation of the *β*_3_-AR gene, resulting in the replacement of tryptophan by arginine at codon 64, was associated with early onset of T2DM [97, 98] and with diabetic retinopathy and nephropathy in Japanese T2DM patients [99, 100]. 

The decrease of content and functional activity of *β*_1_-AR in the diabetic heart led to a blunting of chronotropic effects of NE and other *β*-AR agonists, induced the impaired *β*_1_-mediated cardiac stimulation, and led to a decrease of their stimulating effects on cardiac AC activity [85, 92, 94, 101]. There are grounds to believe that the decrease of *β*_1_-AR function in the diabetic heart, especially in the case of prolonged T1DM, led to the prevention of *β*_1_-AR-stimulated apoptosis, which contributes to progressive myocardial dysfunction. It is well known that selective *β*_1_-AR-antagonists block apoptosis in the rat ventricular myocytes, while the blockers of *β*_2_-AR, on the contrary, enhance this process [102]. 

The chronotropic response mediated via *β*_2_-AR was preserved in diabetic rats with 14-week STZ T1DM, which was demonstrated by the absence of difference between the diabetic and the control atria in their chronotropic and inotropic responses to *β*_2_-AR-selective agonist fenoterol [103]. In this case, the maximum chronotropic response to isoproterenol, nonselective *β*-AR agonist, was decreased with no change in pD_2_ value, while in the case of NE acting preferably via *β*_1_-AR both pD_2_ value and maximum chronotropic effect were decreased significantly. The obtained data showed that the preservation of the *β*_2_-AR signaling in the diabetic rats may be a compensatory mechanism triggered by reducing the functional activity of *β*_1_-AR-mediated pathways. Another evidence in favor of compensatory redistribution of *β*_1_-AR and *β*_2_-AR-mediated signaling pathways in the diabetic heart is the fact that in the heart of 14-week-STZ diabetic rats the contents of *β*_1_-AR protein and *β*_1_-AR mRNA were decreased to 35% and 45%, respectively, whereas the decrease of the maximum chronotropic response of the right atria to *β*_1_-AR agonists exceeded 70% of that in control, indicating an increase of the contribution of *β*_2_-AR-signaling to *β*-AR-mediated chronotropic effects in T1DM [86, 103]. In the diabetic heart of STZ rats, there was an increase in expression and functional activity of *β*_3_-AR, resulting in the enhanced negative inotropic effect and the bradycardia. The activation of *β*_3_-AR signaling involving both cAMP- and NO-dependent pathways may be a mechanism of preventing the overstimulation of heart muscle by catecholamines, whose level is increased in diabetic cardiomyopathy [96]. 

It was shown that patients with T1DM had a decreased *β*-adrenergic responsiveness of the heart to isoproterenol infusion [85]. In ventricular cardiomyocytes and papillary muscle isolated from STZ rats, the stimulation influence of *β*-AR agonists on AC was decreased due to the disturbances of the upstream components of this system [101, 104]. In the cardiomyocytes and cardiac membrane preparations from rats with STZ-induced T1DM, the AC stimulating effect of isoproterenol was decreased by 10% to 30% [94, 101, 105]. The abnormalities in the AC system sensitive to *β*-AR agonists were detected either at the level of receptor and its coupling to G_s_ proteins or at the level of downstream cAMP-dependent effector proteins but not at the level of AC, whose functional activity in the diabetic heart remained unchanged. This is indicated by the fact that the basal AC activity and the enzyme stimulation by NaF and forskolin in rats with STZ T1DM did not change [101, 106, 107]. 

It should be noted at this point that in the mesenteric artery of rats with STZ T1DM the changes in the expression and functional activity were identified not only in the downstream components of cAMP-dependent cascades, such as PKA and cAMP-dependent phosphodiesterase, but also in some types of AC [108]. The mRNA expression and the protein content of AC5 and AC6 were significantly lower in the diabetic mesenteric artery, as compared to control, and the stimulation of the basal AC activity by the water-soluble forskolin analog NKH477, a putative AC5 activator, was significantly impaired in the mesenteric arteries from rats with STZ-induced T1DM [108]. According to the recent finding, transgenic overexpression of AC5 in cardiomyocytes of mice improved their baseline cardiac function but impaired the ability of the heart to withstand stress, while a knockout of gene encoding AC5 and the pharmacological inhibitors of the enzyme, on the contrary, protected the heart against cardiac stress and cardiomyopathy induced by T2DM and obesity [109]. It follows that the decrease in activity and the changes in the pattern of AC isoforms are a significant contribution in alterations of the blood flow and of the response of heart to catecholamine stimulation in DM. 

One of the main causes of alterations and abnormalities of *β*_1_-AR signaling in the diabetic heart in T1DM is the selective downregulation of *β*_1_-AR due to a significant increase of the level of NE that has high affinity for *β*_1_-AR. The elevation of cardiac NE concentration and NE turnover and uptake may be due to the increased sympathetic nerve activity in T1DM-induced diabetic cardiomyopathy, as in the case of other cardiovascular diseases [110]. It was shown that exercise training reduced the cardiovascular morbidity and mortality during T1DM due to the normalization of the sympathetic outflow and improvement in the responsiveness of the myocardium to autonomic stimulation, which induced the decrease of circulating level of catecholamines and restored the number of *β*_1_-AR [111]. It should be mentioned, however, that exercise training of rats with mild T1DM induced by moderate doses of STZ (45 mg/kg) did not prevent DM-induced changes in *β*-AR expression but improved the sensitivity of cardiac AC to isoproterenol [106]. At the same time, excessive exercise training accentuated T1DM-induced decrease in the myocardial *β*-AR responsiveness to hormonal stimulation due to a significant decrease of *β*_2_-AR expression without affecting the reduced level of *β*_1_-AR [112]. 

A majority of investigators showed that, as a rule, the alterations and abnormalities in AC sensitivity to *β*-AR agonists in T1DM as well as T2DM were associated with the left ventricle, which is the main causal factor of diabetic cardiomyopathy [95, 111, 113, 114]. However, there are data on the changes in functional activity of AC system in the right atrium; they differ significantly from those in the left ventricle [115]. It was shown that in the right atrium of swine with STZ-induced DM the density of *β*-AR was 14% lower, and the forskolin- and GppNHp-stimulated AC activities were reduced by 34 and 23%, respectively, whereas the basal and isoproterenol-stimulated AC activities as well as the content of G_s_ and G_i_ proteins did not change. Despite these alterations, the stimulation of heart rate with isoproterenol and *β*-AR-dependent signaling pathways remained intact in the right atrium of diabetic swine [115]. 

A significant difference was found in the changes of *β*-AR-mediated signaling in ventricular cardiomyocytes from the heart of STZ rats, depending on the gender of animals [116]. In the isolated cardiomyocytes from diabetic female rats, there was no obvious change of the dose-response curve of AC stimulating effect of isoproterenol, whereas in diabetic male rats, there was a considerable decrease in the maximum response to this hormone. One of the causal factors can be a T1DM-induced significant decrease in androgen levels, which negatively affects the *β*-AR regulation of cardiac function [117]. 

Alongside with the altered *β*-AR signaling, there were changes in *α*-AR-regulated signaling pathways in the diabetic heart. In T1DM, the hormonal sensitivity of myocardial *α*-ARs was increased, but the overall number of *α*-AR binding sites was decreased without significant changes in the affinity constants [104, 118–120]. It was shown that, compared with control, the administration of selective *α*-AR agonists to acute diabetic animals led to a more pronounced *α*-AR-mediated responses of the myocardium, such as maximal change in pressure over time and developed tension [121–123]. The study of rats with one-, four-, and 10-week T1DM demonstrated that the changes of *α*-AR-dependent cardiac functions were mainly associated with the alterations in the *α*_1_-AR-mediated pathways involving G_q_ proteins and phospholipase C, while the AC signaling cascades regulated by *α*_2_-AR agonists via G_i/o_ proteins were changed to a lesser extent [124]. At the same time, prolonged T1DM induced by the treatment of animals with dropping doses of STZ (30–40 mg/kg) led to a weakening of the inhibitory effect of NE on forskolin-stimulated AC activity in the myocardial membranes and to a decrease of NE-induced stimulation of G_i_ protein GppNHp-binding capacity [32]. 

We detected a significant decrease of the AC inhibitory effect of somatostatin acting via G_i_-coupled SSTRs [32]. There are all reasons to suppose that the decrease of regulatory influence of somatostatin on the AC system is associated with the decreased function of G_i_ proteins, as well as with the reduced number of SSTRs, like in the diabetic atria with markedly reduced levels of SST_2_R mRNA and protein [125]. In the atria of STZ-treated rats, the inhibitory effect of somatostatin on atrial natriuretic peptide secretion was attenuated compared to that in the control atria. It means that somatostatin-regulated cAMP signaling is likely to be involved in the pathogenesis of cardiovascular complications of T1DM. 

The data on the weakening of G_i_-coupled AC cascades regulated by different hormones indicate that the functions of G_i_ proteins in the heart of animals with T1DM, the same as in the brain, are highly attenuated. This assumption is supported by the findings of other authors who showed the decrease of G_i_ proteins functions in the diabetic heart and no significant changes of G_s_ proteins expression and activity [32, 92, 101]. As early as 1994, it was shown that in the cardiomyocytes isolated from STZ rats the expression of G_i2_ and G_o_ proteins was reduced by 58% and 27%, respectively, whereas the expression of G_s_ proteins did not change [101]. A significant decrease in the level of G_i_, but not G_s_ proteins, was identified in the heart of rats with 4–6-week STZ DM [92]. The decrease of the expression of G*α*_i_ subunit and functional activity of G_i_ proteins was identified in the aorta of rats with short-term T1DM and in the aorta exposed to high glucose, without any changes in the levels and functions of G_s_ *α* subunit [126, 127]. The expression of G*α*_i2_ and G*α*_i3_ subunits as determined by immunoblotting techniques was reduced by about 40% three days after STZ treatment and decreased by about 70% and 50%, respectively, 5 days after the treatment. In the diabetic aorta, the inhibitory effects of GTP*γ*S and hormonal agents, such as angiotensin II, oxotremorine, and atrial natriuretic peptide analogs, on forskolin-stimulated AC activity were attenuated compared with control, which points to the decrease of receptor-independent and -dependent functions of G_i_ proteins [126]. The decrease of the expression of the G*α*_i2_ and G*α*_i3_ subunits and G_i_-mediated AC signaling in the case of hyperglycemia induced by short-term STZ DM or high concentration of glucose (above 20 mM) may be attributed to the increase of O_2_^−^ and ONOO^−^ levels, since the treatment with antioxidants reversed the hyperglycemia-induced abnormalities in AC system to control [128]. It should be mentioned that the oxidative stress enhanced in T1DM and acute hyperglycemia is one of the key causes of the increase of the expression of G_q/11_ *α* subunits and strengthening of the phospholipase C*β* signaling [129]. 

In experimental and human T2DM, the responsiveness of the myocardium to *β*-AR stimulation and AC stimulating effects of *β*-AR agonists was, as a rule, decreased, and, unlike in T1DM, the number of *β*-ARs did not differ significantly from control [41, 130–132]. As far as alterations in AC sensitivity to agonists are concerned, they depend on the model of T2DM, its duration and severity. In the myocardium of eight-month-old rats with the neonatal model of T2DM, the AC stimulating effect of *β*-agonist isoproterenol was increased, but not very much, and in the same tissue of 18-month-old diabetic rats, it was, on the contrary, reduced significantly compared with the respective control. The AC stimulating effect of relaxin, an insulin-like peptide, in the heart of rats with long-term neonatal T2DM was reduced to a high extent, and in 18-month-old rats it did not exceed 46% of that in the respective control. The AC inhibitory effect of NE on forskolin-stimulated AC activity in the myocardial membranes of eight- and 18-month-old diabetic rats was, at most, 51% and 46% of that in control. These findings suggest a significant decrease of the sensitivity of cardiac AC to adrenergic agonists and relaxin in very prolonged neonatal T2DM [41]. 

There were no significant differences between the basal and forskolin-stimulated AC activities in the heart of control rats, on the one hand, and diabetic animals with prolonged neonatal STZ T2DM and Zucker *fa/fa* rats. On the other hand, GppNHp-stimulated AC activity was reduced in the diabetic heart, especially in the case of the neonatal model [33, 41, 132]. These data indicate a weakening of the G_s_ protein function with no change in the catalytic activity of the enzyme. One of the causes of the decline of G_s_ proteins activity is the T2DM-induced hyperhomocysteinemia that leads to the attenuation of G_s_ proteins activity and the decrease of the number of *β*_2_-ARs, as shown in *db/db* mice [133]. A decrease of homocysteine level induced by the physical exercises led to the restoration of *β*_2_-AR-G_s_ protein-AC system in the diabetic heart. 

## 4. cAMP Signaling in the Skeletal Muscles

The first data on the functional state of AC system in the skeletal muscles was obtained by Garber in 1980 [134]. It was shown that in the skeletal muscles of rats with STZ-induced T1DM epinephrine-stimulated AC activity was reduced by 60%, while the basal and the NaF- and 5-HT-stimulated AC activities did not change. Later, it was found that the density of *β*-ARs and AC stimulating effect of *β*-AR agonists, reduced in different types of the skeletal muscles, for example, the soleus muscles and the vastus lateralis muscles, of rats with STZ DM, were partially restored by physical training that was a progressive 10-week treadmill running program [135, 136]. We showed that in the skeletal muscles of rats with 30-day STZ T1DM the stimulating effect of *β*-adrenergic agonists and the inhibitory effects of NE and 5-HT on AC activity were reduced, as compared with control [31, 137]. The increase of GppNHp binding capacity of G proteins induced by adrenergic agonists and 5-HT in the sarcolemmal membranes of diabetic animals was lower than in control, and the difference between the diabetic and control rats was most pronounced in the case of G_i_ proteins. A special mention deserves the fact that the acute hyperglycemia caused by very short STZ T1DM also led to a decrease of functional activity of G_i_-coupled AC signaling cascades, as it follows from a significant weakening of inhibition of forskolin-stimulated AC activity and stimulation of GppNHp binding capacity of G_i_ proteins induced by somatostatin in the skeletal muscles of rats with one-day T1DM [36]. 

The deficiency of G_i_ proteins in the skeletal muscles in T1DM not only induced the abnormalities in the cAMP signaling, but also it reduced the sensitivity of the muscles to insulin action. It was shown that the expression of constitutively activated mutant form of the G*α*_i2_-subunit with the replacement of Asn^205^ by Leu in the skeletal muscles, liver, and the adipose tissue of mice with STZ-induced T1DM markedly ameliorated glucose tolerance and fasting glucose levels and, in addition, restored glycogen synthase activity in the tissues of transgenic mice [138]. The above is due to the fact that the G*α*_i2_-subunit is involved in the regulation of the translocation of insulin-sensitive glucose transporter GLUT4, and in the *in vivo* conditions the expression of Q^205^L G*α*_i2_-subunit led to the increase of glucose transport and GLUT4 translocation in the skeletal muscles even in the absence of insulin stimulation [139]. 

The data on the functioning of the AC system in the skeletal muscles in T2DM are rare. There is a report that in the skeletal muscles of young *db/db* mice the epinephrine-induced lipolytic activation was reduced, but the stimulating effects of this hormone on AC and glycogen phosphorylase activities were unaltered [140]. This gives evidence for the impairment of epinephrine-induced lipolytic activation in the skeletal muscle of *db/db* mice at the level of components of cAMP-dependent signaling cascades, downstream of AC. 

## 5. cAMP Signaling in the Liver

As early as 1984, it was found that in the hepatocytes of rats with STZ T1DM glucagon-mediated regulation of cAMP formation was deranged owing to a combined decrease of glucagon receptors, impairment of their coupling with AC, and altered basal AC activity [141]. The basal and NaF-, GppNHp-, Mn^2+^-, and glucagon-stimulated AC activities in the hepatic plasma membranes isolated from the diabetic liver were reduced by 50%, and the content of G_s_-coupled glucagon receptors was decreased by 67%, all these changes being partially reversed by insulin treatment. 

Later, it was shown that, in the diabetic liver, G_i_-coupled signaling cascades, cAMP dependent in particular, underwent significant changes, and these alterations were mainly associated with reduced activity of G*α*_i_ subunits, preferably G*α*_i2_ [142–144]. The decrease of G_i_ proteins function was due to decreased expression of G*α*_i_ subunits and their increased phosphorylation, depriving G_i_ proteins of the ability to participate in signal transduction. The levels of G*α*_i2_ and G*α*_i3_ subunits in the hepatocyte plasma membranes were decreased significantly, but the level of G*α*_s_ subunits was increased. However, in the whole liver, there were no detectable changes in the levels of G*α*_i2_ and G*α*_i3_ subunits, but only the decrease of transcription level of G*α*_i2_, but not of G*α*_i3_, subunit was found [142]. These differences may be related to the fact that DM-induced changes in parenchymal cells (hepatocytes) constituting only 60% of cells in the liver may have been masked by the G protein complement of the other, nonparenchymal, cells. It was shown that STZ-induced T1DM caused a profound increase in the steady-state level of G*α*_i2_ subunit phosphorylation in hepatocytes. G*α*_i2_ subunit in hepatocytes from diabetic animals was phosphorylated exclusively at the protein kinase C site but not observed at the PKA phosphorylation site. This reflected an augmentation of the protein kinase C signalling cascades in hepatocytes from STZ-treated rats due to an increased protein kinase C activity and a reduction in protein phosphatases activity [144]. The weakening of G_i_-mediated signaling was associated with the increase of the signaling cascades involving G_s_ proteins and the enhancement of catalytic function of AC. 

The influence of STZ T1DM on the basal and hormone-sensitive AC activity and the binding of G_s_/G_i_-coupled receptors in the hepatic membranes directly depended on the duration and severity of the diabetic state [145]. No changes in the basal and GppNHp-, Mn^2+^-, glucagon-, and isoproterenol-stimulated AC activities and the binding affinity and capacity of *β*-AR were observed within 3 days after STZ treatment. Fifteen days after the treatment, AC stimulation by hormonal and nonhormonal agents and the binding capacity of *β*-AR were greatly decreased, as compared to control. The amount of *α*-ARs was also reduced in the hepatic membranes isolated from rats with 15-day STZ T1DM, while it did not change in 3-day diabetic animals [145]. These results may explain the considerable differences in AC signaling detected in the liver of diabetic animals and emphasize the importance to study the dynamics of changes in the AC system when the disease is still developing. 

The alterations of AC signaling, hormonal sensitivity of *β*-ARs, and G protein pattern were detected in the liver in the genetic models of T2DM. In the liver membranes isolated from *ob/ob* mice with hyperglycemia, hyperinsulinemia, and obesity typical of T2DM, the number of *β*_2_-AR binding sites was increased threefold, and the response of AC to catecholamines was significantly enhanced as compared to both control animals and *db/db* mice, the other animal model of T2DM [146]. The content of G_i_ and G_s_ proteins, especially their G*α*_i2_ subunits, was reduced in the liver of *ob/ob* and *db/db* mice [146–148]. In the hepatic membranes of *db/db* mice, the levels of expression of G*α*_i2_, G*α*_i3_, and G*β* subunits were reduced by 75%, 63%, and 73%, respectively, and the maximal inhibitory effect of GppNHp on forskolin-stimulated AC activity was reduced to 60%, as compared to lean animals, which indicates the abolition of G_i_ proteins activity in the liver in T2DM [149]. Regrettably, the data on the lack of significant changes in the liver of STZ rats and genetically diabetic mice is very scanty. It may be probably due to the difference in the DM models, duration, and severity of the disease and to the application of different approaches in the study of G_i_ protein functions. For instance, in the early studies, there were no significant differences in the functional activity of G_i_ proteins in the membranes isolated from hepatocytes and the whole liver of the diabetic animals, such as fat Zucker (*fa/fa*) rats and rats with short, one-week, STZ T1DM [150, 151]. 

## 6. cAMP Signaling in the Adipose Tissue

The adipose tissue plays a pivotal role in the whole body energy homeostasis. The functioning of adipocytes is regulated via cAMP-dependent signaling pathways, some of which are canonical PKA-dependent pathways, while the others are carried out through the novel exchange protein activated by cAMP (Epac) [152]. The deregulation of these signaling cascades and their cross-talk with insulin signaling in adipocytes leads to the alterations in energy expenditure and/or feed efficiency and results in obesity and T2DM. 

Regulation of metabolism in the adipose tissue has been shown to be impaired in genetically obese animals with hallmarks of developing T2DM, and the important role in these pathological changes belongs to adrenergic signaling [153]. As is well known, *β*-AR agonists stimulate AC activity via G_s_-coupled *β*-ARs and enhance lipolysis in the adipose tissue. It was found that adipocytes from genetically obese *ob/ob* C57BL/6J mouse and +/+ C57BL/6J mouse fed on a high fat diet for 16 weeks and Zucker *fa/fa* rats displayed an impaired response of AC to stimulation by *β*-AR agonists due to a significant decrease of *β*_1_-AR and, especially, *β*_3_-AR expression [153–159]. The decrease of *β*_3_-AR and *β*_1_-AR expression was found in the white and brown adipose tissues from 12-week-old *ob/ob* mice, whereas *β*_2_-AR mRNA level did not change significantly [155, 158]. As a consequence, in obese animals, the stimulation of AC activity by selective *β*_3_-AR agonist CL316.243 was decreased by 75% in the epididymal white adipose tissue and by 90% in the brown adipose tissue, and the corresponding AC effects of *β*_1_/*β*_2_-AR agonist epinephrine were also decreased considerably, though to a lesser extent compared with those of CL316.243 [153]. The maximal isoproterenol-stimulated lipolysis in the adipocytes from ZDF rats was significantly lower, as compared to control animals, and the EC_50_ values for isoproterenol to increase lipolysis in the adipocytes from normal and obese rats were 2 and 7 nM, respectively [159]. These data demonstrate the significant decrease of *β*-AR-mediated lipolytic activity in the adipose tissue of animals with T2DM-like metabolic disorders. In this connection, the assumption was put forward concerning alterations of the sensitivity of AC in the adipose tissue in T2DM to other activators of lipolysis, such as GLP-1, glucagon, and glucose-dependent insulinotropic polypeptide-1 [160]. But this question is still open. Moreover, recently it was shown that the regulation of lipolysis by glucagon at the physiological concentrations (below 0.1 nM) was carried out via cAMP-independent signaling mechanisms [161]. 

It was established some time ago that endogenous adenosine in the adipocytes tonically activates G_i_-coupled adenosine receptor of the type 1, highly expressed in the adipose tissue, reduces AC activity and cAMP content, and, the same as insulin, inhibits lipolysis. As a result, the plasma free fatty acid concentration elevated in metabolic syndrome, and T2DM decreases; this fact may be of use in the treatment of these disorders. It was found that in the adipose tissue of ZDF and normal rats the antilipolytic activity of CVT-3619, a partial agonist of the type 1 adenosine receptor, and IC_50_ values of CVT-3619-induced inhibition of free fatty acid release from adipocytes were similar [159]. In addition, CVT-3619 is a stronger inhibitor of lipolysis than insulin, especially in the adipocytes isolated from ZDF rats resistant to the antilipolytic action of insulin. It seems reasonable to suppose that the maintenance of the activity of G_i_-coupled purinergic system responsible for antilipolytic activity can partially compensate for a considerable attenuation of insulin antilipolytic effect in the adipose tissue of rats with insulin resistance [162]. 

In the adipose tissue of STZ diabetic rats, G_i_ proteins functions were decreased, but G_i_-mediated signaling was, on the contrary, unaltered or even strengthened [163]. The significant weakening of functional activity of G_i_ proteins was manifested as decreased inhibitory effects of low concentrations of GppNHp on forskolin-stimulated AC activity and high concentrations of GTP on isoproterenol-stimulated AC activity. The AC inhibitory effects of N^6^-(phenylisopropyl)adenosine, nonmetabolizable adenosine analogue, prostaglandin E_2_, and nicotinate were either unchanged or even apparently more effective in the adipocyte membranes from diabetic animals [163]. 

Since insulin-mediated glucose uptake is highly sensitive to the levels of the facilitative glucose transporter protein GLUT4, repression of GLUT4 expression correlated with insulin resistance in the adipose tissue [164]. The differentiation-dependent GLUT4 transcription was under control of class II histone deacetylases and regulated by adrenergic agonists that generally increase intracellular cAMP level and, as a result, promoted the association of histone deacetylases with the GLUT4 promoter, which led to the decrease of GLUT4 transcription. The cAMP-dependent recruitment of histone deacetylases to the GLUT4 promoter was dependent on the GLUT4 liver X receptor (LXR) binding site, so that the activation of LXR signaling prevented the loss of GLUT4 expression in DM and obesity [164]. This finding offered a new understanding of how cAMP-signaling pathways regulated by adrenergic agonists alter gene expression in adipose tissue in DM. 

## 7. Conclusion

This review presents the data concerning the changes in AC signaling system sensitive to biogenic amines, peptide hormones, and purines in DM and its complications, such as diabetic encephalopathy, diabetic peripheral neuropathy, diabetic cardiomyopathy, and others. The main causes of the changes are hyperglycemia, lipidemia, oxidative stress, and other metabolic abnormalities typical of DM, as well as alterations in the central and peripheral signaling cascades induced by the resistance of the cells and tissues to the regulatory action of insulin/IGF-1 and leptin as well as by relative or absolute insulin deficiency. These changes largely depend on the type and duration of DM and on the model of the disease in the case of experimental DM. The degree of alterations and abnormalities in the AC signaling system correlates very well with the severity of the DM and its complications. The changes in AC signaling at the early stages of DM are compensatory and, as a rule, reversible, while at the later stages of the disease, when it is accompanied by many complications, these changes occurring in some particular AC signaling cascades induce the alterations in many signaling pathways, both cAMP dependent and independent, which leads to the disruption and unbalance of global integrated signaling network and, as a result, these changes become irreversible. The most complex pattern and dynamics of the changes of the AC system are in the brain, where they involve a large number of neurotransmitters and signaling cascades regulated by them and are region and cell specific. To know the origin and reversibility of changes in hormonal signaling in DM is very important for the development of effective strategy in diagnostics and treatment of this disease and its complications. 

One of the most important questions here is whether the abnormalities in DM occur in the tissue or organ in the AC system sensitive to one or a limited number of the related hormones; if so, how they cover the other signaling cascades, or whether at the initial stage of the disease they occur independent of each other in multiple AC signaling pathways. The latter seems more likely since a few days after T1DM induction by STZ treatment the alterations are detected in multiple AC cascades regulated by various hormones and involving both G_s_ and G_i_ proteins. The same applies to cAMP-independent pathways. Along with this, there are pieces evidence that at the initial stages of DM or in prediabetes the disturbances occur only in one or a small number of AC signaling cascades, while the others remain unchanged. Then the alterations in one signaling cascade can induce, according to the “domino” principle, changes in the others. The domino principle is well illustrated by the fact that the disturbances in some particular signaling cascades of the brain cause numerous abnormalities in the neuronal network, which may lead to insulin resistance or insulin deficiency and, as a result, to the development of DM and its central and peripheral complications [12, 50, 52, 59, 60, 165–169]. 

Summing up, to identify the initial hot spots in AC signaling, the abnormalities of which lead to an avalanche of hormonal disturbances, we must know the etiology and pathogenesis of DM and its prevention at the stage of prediabetic state, such as obesity and metabolic syndrome in T2DM and autoimmune damage of pancreatic *β*-cells in T1DM. The causal relation between DM and the altered hormone-sensitive AC system is not a one-way avenue, from DM-induced alterations of hormonal signaling in CNS and peripheral organs; the alterations in AC signaling pathways may be a causal factor of DM and its complications. This speaks in favor of the use on a wide scale—not only in the treatment but also in prevention of DM—of hormonal and nonhormonal agents that control functional activity of signal proteins, the components of the AC system, and have an influence on availability, transport, and secretion of hormonal molecules. Among them are serotonin, D_2_-agonist bromocriptine, MC_3_R and MC_4_R agonists, the regulators of AC activity, and drugs controlling their uptake and transport, selective serotonin reuptake inhibitors in particular [35, 170–173]. Note that these agents may be useful for T2DM patients with resistance to typical antidiabetic drugs metformin and sulfonylurea, as it is shown in the case of bromocriptine therapy [174, 175].

## Abbreviations

AC: Adenylyl cyclase
AgRP: Agouti-related peptide
AR: Adrenergic receptor
DA: Dopamine
DAR: Dopamine receptor
DM: Diabetes mellitus
GLP-1: Glucagon-like peptide-1
G_s_ and G_i_ proteins: Heterotrimeric G proteins of the stimulating and inhibitory types
GPCR: G protein-coupled receptor
GppNHp: 5′ Guanylylimidodiphosphate
5-HT: 5-Hydroxytryptamine
5-HTR: 5-Hydroxytryptamine receptor
IGF-1: Insulin-like growth factor-1
MCR: Melanocortin receptor
*α*-MSH: *α*-Melanocyte-stimulating hormone
NE: Norepinephrine
PACAP: Pituitary adenylyl cyclase-activating polypeptide
PKA: Protein kinase A
SSTR: Somatostatin receptor
STZ: Streptozotocin
T1DM and T2DM: Types 1 and 2 diabetes mellitus.
